# Posterior Circulation Stroke Secondary to Basilar Artery Thrombosis With a Fatal Outcome

**DOI:** 10.7759/cureus.34146

**Published:** 2023-01-24

**Authors:** Gajanan N Umalkar, Gajanan Chavan, Charuta Gadkari, Mayur B Wanjari

**Affiliations:** 1 Department of Emergency Medicine, Jawaharlal Nehru Medical College, Datta Meghe Institute of Higher Education and Research, Wardha, IND; 2 Department of Research and Development, Jawaharlal Nehru Medical College, Datta Meghe Institute of Higher Education and Research, Wardha, IND

**Keywords:** pcs (posterior circulation stroke), gcs (glasgow coma score), basilar artery, thrombectomy, stroke

## Abstract

Stroke is a regularly encountered emergency by emergency physicians, categorized based on the culprit artery and diagnosed based on non-contrast computerized tomography (CT) brain, which is supported by clinical examination that can be treated intravenously by thrombolytic agents or mechanical thrombectomy. Here we present one such case, which was brought to the emergency room with symptoms of posterior circulation stroke within 8 hours and underwent mechanical thrombectomy.

## Introduction

A pair of vertebral arteries (VA), distal vertebral, and basilar are intracranial arteries. These arteries together supply the brainstem, cerebellum, medial and postero-lateral thalamus, occipital lobes, and occasionally sections of the medial temporal and parietal lobes [1,2]. Posterior circulation strokes (PCS) account for around 20-25% of all ischemic strokes [3]. PCS is a clinical entity associated with ischemia related to stenosis, thrombosis, and embolism in the posterior circulation arteries (vertebral, basilar, and other intracranial vessels) and their branches [4]. According to the trial of ORG 10172 in acute stroke treatment (TOAST), strokes are classified as (a) cardioembolic, (b) large vessel disease, (c) small vessel disease, (d) stroke of undetermined significance, and (e) a stroke of determined significance or rare and minor vessel diseases [5]. Diagnosis can be difficult because presenting symptoms are atypical. Despite advances in imaging technology, life-threatening diseases like basilar artery occlusions are frequently misdiagnosed and hence go untreated, resulting in mortality. Hence we present a patient who was brought to the emergency room in an unconscious state with a lower Glasgow Coma Scale (GCS) and underwent a mechanical thrombectomy with a fatal outcome. As a result, early detection of symptoms and causes of posterior circulation ischemia is critical for choosing the most appropriate treatment approach [6].

## Case presentation

**Figure 1:**
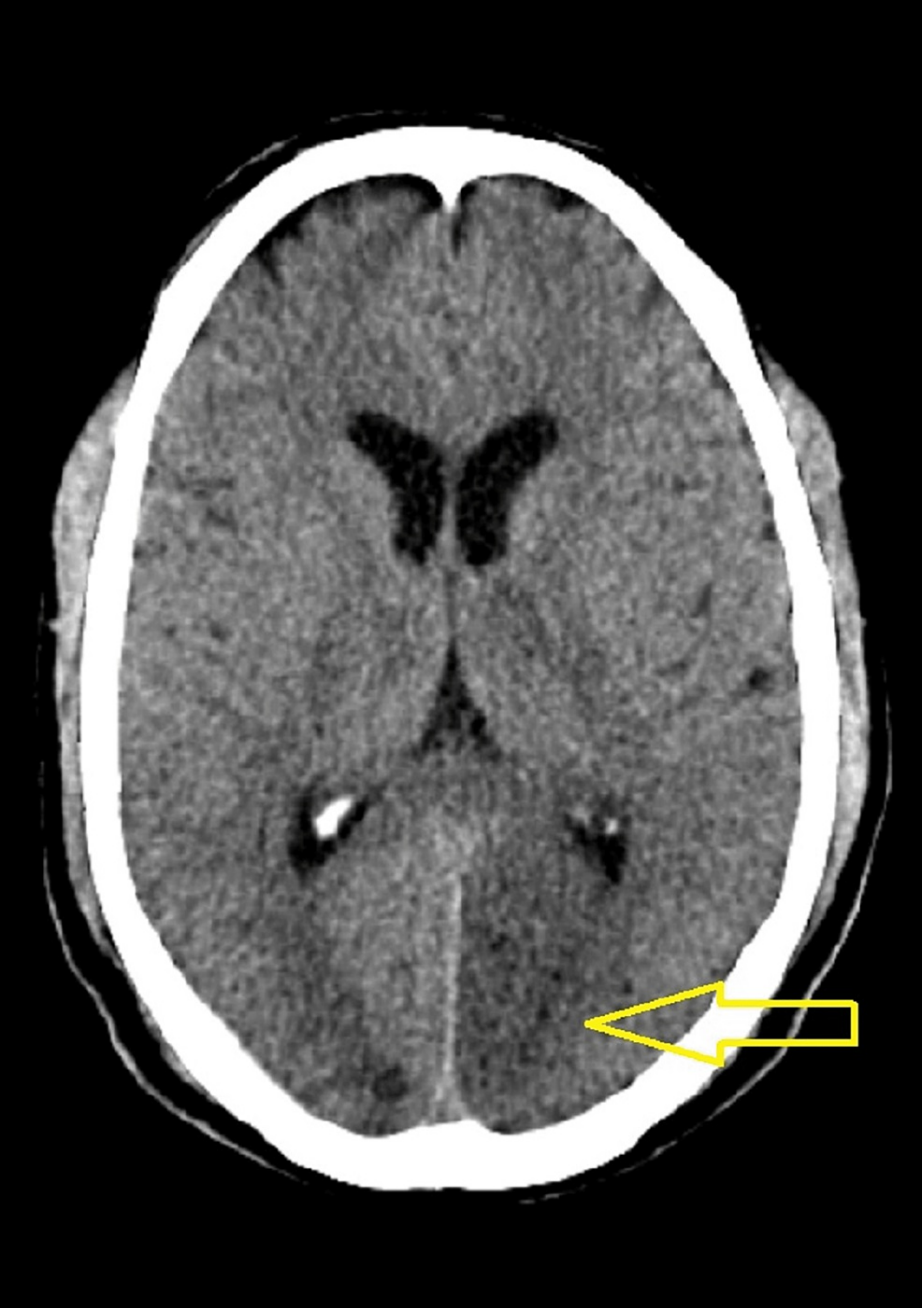
Acute infarct in the left occipital lobe of the cerebrum and right thalamus

A 70-year-old man was brought to the emergency room in an unconscious state with a history of blurring of vision and giddiness for 4 hours which was sudden in onset while the patient was sitting in a chair at his home, associated with multiple episodes of vomiting for 7 nonhours - projectile in nature containing food particles. On a primary survey, his airway was threatened. Saturation was 90% on room air, and respiratory rate was 20/minute. His pulse rate was 100 beats per minute, and his blood pressure of 170/100 mm hg. The patient random blood sugar level was 184 mg/dl. A 12 lead electrocardiogram (ECG) was done, which was suggestive of sinus rhythm. The patient was intubated with rapid sequence intubation with a cuffed endotracheal tube because of low GCS and was attached to a mechanical ventilator (volume control mode) under complete sedation and paralysis. He has had a history of hypertension for 10 years and is on oral telmisartan 40 mg once a day. There was no history of seizures, falls, trauma, diabetes mellitus, bronchial asthma, or previous history of stroke or ischemic heart disease. On systemic examination patient's GCS was E1V1M4, pupils were unequally reactive to light, Babinski sign was positive on the right side, meningeal signs were absent, and his National Institutes of Health Stroke Scale (NIHSS) score was 24. An emergent non-contrast head computed tomography (CT) was obtained that was concerning for ischemic stroke, as shown in (Figure 1).

**Figure 2:**
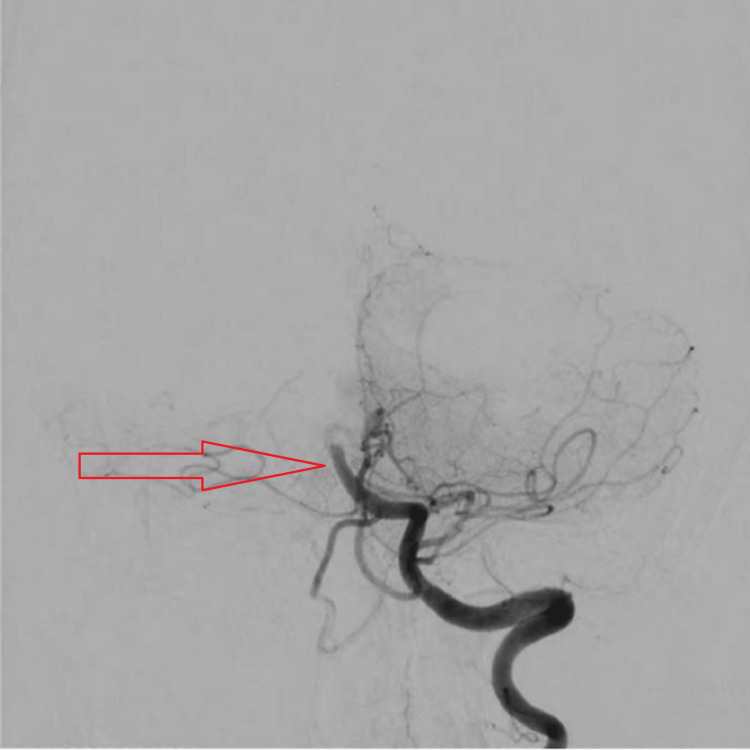
Four vessel DSA: complete occlusion of the basilar artery DSA: digital subtraction angiography

The patient was shifted to Cathlab immediately for four vessels digital subtraction angiography (DSA). Scan indicating complete occlusion of the basilar artery as shown in (Figure 2) as magnetic resonance imaging (MRI) was contraindicated in the patient because of the metallic implant in the right arm.

**Figure 3:**
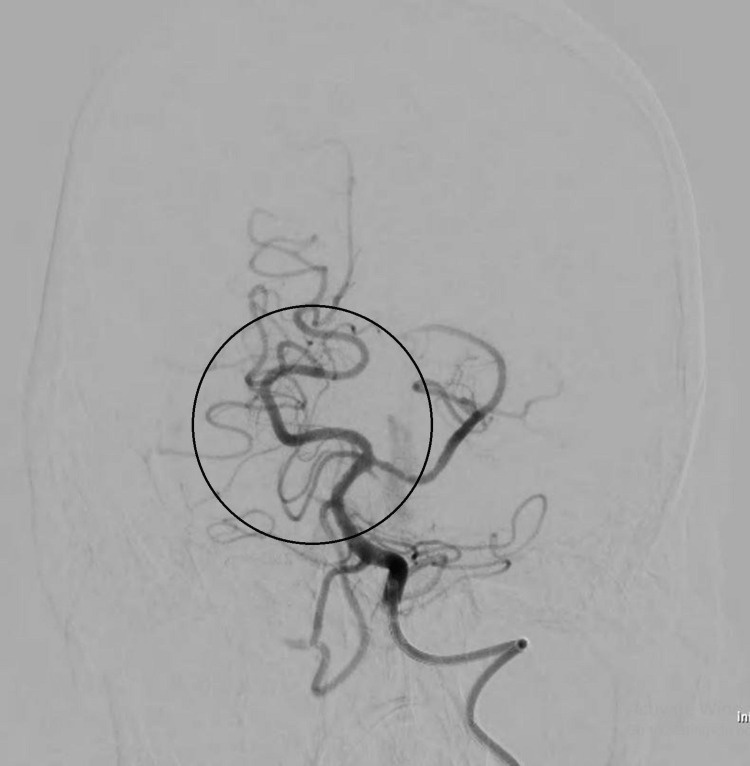
Post-thrombectomy: recanalization of the basilar artery

Mechanical thrombectomy was done using Clot retrieval devices Catchmacxi 6 by 50 mm, and prior 4 by 40 mm was done, as shown in (Figure 3).

The post-procedure basilar artery was recanalized, as shown in Figure 3. The patient was shifted to the intensive care unit (ICU), where 2D echocardiography was done within normal limits. The patient was managed with intravenous injection (INJ) of mannitol 1gm/kg, levetiracetam 1gm twice a day, intravenous (IV) fluid normal saline 80 ml/hr, and low molecular weight heparin 40 mg subcutaneous (SC) once a day (deep vein thrombosis (DVT) prophylaxis), tab aspirin 75 mg and clopidogrel 75 and rosuvastatin 20 mg at night through Ryle's tube. Despite early intervention, there was a fatal outcome.

## Discussion

Vertebrobasilar insufficiency is a disorder that affects the posterior circulation and is caused by ischemia-related blockage of the posterior circulation arteries and their branches. Risk factors such as hypertension, smoking, hypercholesterolemia, atrial fibrillation, and coronary artery disease leading to an increased risk of stroke [7].

Hypoperfusion to the brainstem, cerebellum, and occipital cortex results in vertigo, diplopia, dysarthria, dysphagia, disequilibrium, ataxia, and visual field defects. Depending on the location of the blockage, basilar artery occlusion, which accounts for 1% of all strokes, can lead to the brainstem or thalamic infarction, which may cause severely diminished consciousness, coma, and cardiorespiratory abnormalities. Even 24 hours after the beginning of symptoms, mechanical thrombectomy (clot retrieval) can be beneficial [8].

In this case, the patient was brought unconscious; hence, vascular pathology was suspected, confirmed by CT brain. Based on clinical examination and history given by relatives of the patient other causes of vertigo were not considered. Our case presentation is similar to Mario Tortora et al. [9].

## Conclusions

A large number of patients approach the emergency room with complaints of giddiness and loss of balance frequently. This patient should be given special attention until the stroke is ruled out. Despite arriving out of the IV thrombolysis window time, this patient needs to be evaluated immediately. They should be sent for a scan because mechanical thrombectomy for PCS can be performed for up to 24 hours, decreasing overall mortality and improving outcomes.
